# Fetuin-a expression profile in mouse and human adipose tissue

**DOI:** 10.1186/s12944-020-01225-7

**Published:** 2020-03-12

**Authors:** Abdelkrim Khadir, Sina Kavalakatt, Dhanya Madhu, Ali Tiss

**Affiliations:** 1 Genetics and Bioinformatics department, Research Division, Dasman Diabetes Institute, Kuwait City, Kuwait; 2 Biochemistry and Molecular Biology department, Research Division, Dasman Diabetes Institute, P.O. Box1180, 15462 Dasman, Kuwait

**Keywords:** Fetuin-a, Adipose tissue, Human adipose primary cells, Mouse adipose tissue

## Abstract

Fetuin-A (Fet-A) was one of the first hepatokines to be reportedly linked to metabolic diseases. Fet-A was also suggested to be an adipokine, but its expression in the adipose tissue remains debatable. Here we compared the expression of Fet-A between human and mice adipose tissue biopsies as well as among human subcutaneous tissue and visceral adipose tissue primary cells, and mouse 3 T3-L1 cells at various stages of differentiation. Fet-A was expressed in mice biopsies and cells but not in human biopsies and cells, except in visceral adipose tissue primary cells following differentiation. Although the marginal expression of Fet-A in human visceral adipose tissue, a major contribution of Fet-A expression in human adipose tissue to systemic Fet-A levels is discounted, but it could indicate specific local Fet-A action in the visceral adipose tissue.

## Main text

The hepatokine fetuin-A (Fet-A) is linked to obesity and type 2 diabetes, but the causality of the association was not supported by a recent Mendelian study in a global population [1]. Fet-A is a natural inhibitor of insulin receptor tyrosine kinase and contributes to insulin resistance in rodents and humans [2, 3]. In humans, high Fet-A levels are linked to obesity, metabolic syndromes, and diabetes [4]. Recent studies suggested that Fet-A is an adipokine; however, its expression in adipose tissue remains unclear depending on the type of cells and species investigated [5, 6]. In a recent study, we assessed Fet-A levels in plasma and human subcutaneous adipose tissue (hSAT) of obese adults with and without diabetes [6] and found that Fet-A protein levels in hSAT were significantly increased in obese adults with diabetes. We suggested that Fet-A was present in hSAT and peripheral blood mononuclear cells (PBMCs) as a result of its uptake from circulation rather than its endogenous expression because Fet-A mRNA was detected neither in hSAT nor in PBMCs. In our proposed model, hSAT would be a reservoir for Fet-A. However, we do not exclude the possibility that hSAT marginally contributes to Fet-A production and has a potentially limited paracrine and/or autocrine function [6]. Jialal and Pahwa recently commented on our study and expressed concerns regarding the lack of Fet-A expression in hSAT [5].

**Fig. 1:**
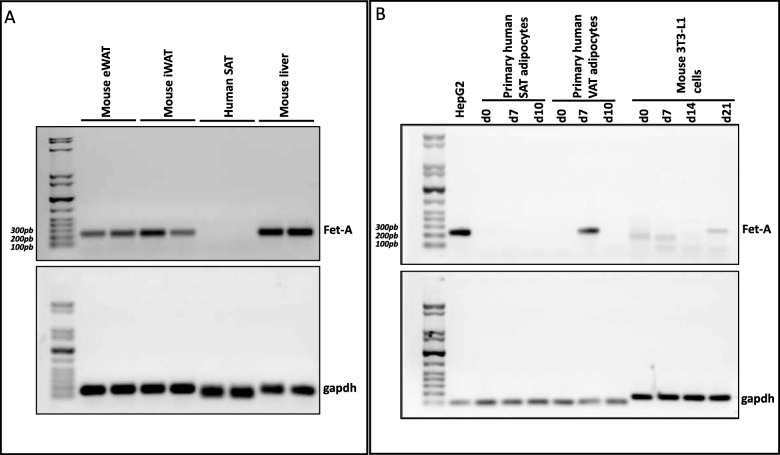
Fetuin-A expression in human and mouse adipose tissue and adipocytes. **a** Total RNA was isolated from human and mouse tissue biopsies and subjected to RT-PCR and agarose gel analysis. **b** Total RNA was isolated from human primary subcutaneous adipose tissue and visceral adipose tissue adipocytes and mouse 3 T3-L1 cells at various stages of differentiation and subjected to RT-PCR and agarose gel analysis. 15 ng of PCR products were loaded on the agarose gel for all samples, excluding mouse liver and HepG2 cell positive controls, for which only 5 ng were loaded. The gels represent three independent experiments

In an attempt to address these concerns, we analyzed and compared the expression of Fet-A in hSAT, epididymal visceral mouse adipose tissue (eWAT), and inguinal mouse adipose tissue (iWAT) biopsies using RT-PCR. Additionally, we assessed Fet-A expression during the differentiation of human primary preadipocytes from hSAT, human visceral adipose tissue (hVAT), and mice 3 T3-L1 cells. Our results revealed high Fet-A expression in both mice biopsies; however, we did not detect any Fet-A transcripts in hSAT (Fig. 1a), which was consistent with the findings of previous reports, including ours [6–8].

Remarkably, when we differentiated human preadipocytes, we did not detect any Fet-A transcripts in the hSAT preadipocytes, hVAT preadipocytes, or hSAT adipocytes; however, Fet-A transcripts were present in the late stages of differentiation in hVAT adipocytes (Fig. 1b). This observation was further confirmed at Fet-A protein levels (data not shown). Similarly, Hennig et al. previously reported the absence of Fet-A mRNA in hSAT preadipocytes [8]. In our previous study, we reported the absence of Fet-A mRNA in 3 T3-L1 preadipocytes and differentiated adipocytes at day 8 [6]. Based on the suggestion by Jialal and Pahwa [5], we differentiated 3 T3-L1 preadipocytes for a longer period and detected faint Fet-A transcript at day 21 at the anticipated size. As previously reported, we also detected faint bands at days 0 and 7, although with reduced sizes compared with the positive control (Fig. 1b). Nevertheless, sequencing the PCR products at various differentiation days, including day 21, did not match with Fet-A sequence (data not shown). It is essential to stress that Fet-A transcripts in hVAT and 3 T3-L1 differentiated adipocytes were only detected at high PCR amplification cycles compared with the positive controls, including HepG2, mouse liver, and GAPDH (32, 35 versus 17, 18 and 16 cycles, respectively). The difference in amplification (15–17 cycles) reflects a difference of several thousand-fold less expression of Fet-A in differentiated hVAT and 3 T3-L1 adipocytes. The low level of expression in tissues is often considered Fet-A negative. For instance, Jahnen-Dechent et al. detected Fet-A mRNA in Fet-A knockout mice despite the mice having been shown to lack *Fet-A AHSG* [9]. Furthermore, Song et al. estimated the expression ratio of Fet-A in human and mice liver compared with their respective adipose tissues and reported ratios of 7314-fold and 86-fold, respectively [10], thus highlighting both the lower levels of Fet-A expression in adipose tissue and the much lower expression levels in human adipose tissue compared with that in mouse adipose tissue.

In conclusion, we confirm the high expression of Fet-A in mouse adipose tissue depots and its absence in hSAT and its primary cells. Fet-A was detected in human primary VAT cells at low levels but only after differentiation. Therefore, the marginal expression of Fet-A in hVAT could indicate Fet-A specific local action and requires further investigation.

## Material and methods

hSAT biopsies were collected from consented adults and processed during our previous study [6]. Mouse biopsies were provided by Dr. R. Ahmad (Dasman Diabetes Institute, Kuwait). All primers used, quantitative RT-PCR, and agarose gel analysis were according to our previous study [6]. Primary preadipocyte cells from hSAT and hVAT (#PT5001 and #PT5005, Lonza, USA) were processed and differentiated according to the supplier’s instructions.

## Data Availability

Not applicable.

## Abbreviations

eWAT: epididymal visceral mouse adipose tissue
Fet-A: Fetuin-A
hSAT: human subcutaneous adipose tissue
hVAT: human visceral adipose tissue
iWAT: inguinal mouse adipose tissue
PBMC: Peripheral blood mononuclear cell
